# Diabetes Prevalence Among Underserved Older Adults in the US: The Case of Native Hawaiians and Pacific Islanders

**DOI:** 10.1093/geroni/igab046.2384

**Published:** 2021-12-17

**Authors:** Sela Panapasa, William Herman, James McNally

**Affiliations:** 1 Research Center for Group Dynamics, Ann Arbor, Michigan, United States; 2 University of Michigan Medical System, Ann Arbor, Michigan, United States; 3 University of Michigan, Ann Arbor, Michigan, United States

## Abstract

The effects of chronic diabetes among older adults in the United States represent ongoing challenges in diagnosis, treatment, comorbidities, amputations, and the increased risk of death. These challenges are made more complicated among underserved populations due to limited access to healthcare, medication costs, and later diagnosis of the condition. These issues are particularly true for the NHPI population, which has high rates of lifetime diabetes, greater levels of poverty, and inadequate health insurance. Useful statistics about diabetes among the NHPI have been difficult to obtain due to their small population size and lack of inclusion in federal health surveys. While early work by Panapasa examined prevalence among NHPI males in California, no reliable measures of diabetes among older NHPI’s existed at the national level. Released in 2017, the 2014 Native Hawaiian Pacific Islander National Health Interview Survey represents the first representative survey of the health and socio-economic correlates for this population, allowing the examination of health conditions such as diabetes at the national level. This presentation will examine the prevalence of diabetes among NHPI’s aged 60 and older and the impacts of this disease on overall health and quality of life. The paper will use the NHPI-NHIS to examine the use and access to diabetic medications and overall access to affordable health care. The paper will examine differences by age group, gender, immigration status, and ethnicity. While we know the overall prevalence of diabetes is high, this paper will offer new information on differentials within the older NHPI population.

